# Preoperative chemoradiotherapy with capecitabine and triweekly oxaliplatin versus capecitabine monotherapy for locally advanced rectal cancer: a propensity-score matched study

**DOI:** 10.1186/s12885-022-09855-z

**Published:** 2022-07-18

**Authors:** Anchuan Li, Tingxuan Huang, Rong Zheng, Pan Chi, Zhihua Li, Xiaozhong Wang, Benhua Xu

**Affiliations:** 1 Department of Radiation Oncology, Fujian Medical University Union Hospital, Xinquan Road 29, Fuzhou, 350001 China; 2 Department of Radiation Oncology, College of Clinical Medicine, Fujian Medical University, Fuzhou, 350001 China; 3 Fujian Key Laboratory of Intelligent Imaging and Precision Radiotherapy for Tumors, Fujian Medical University, Fuzhou, 350001 China; 4 Clinical Research Center for Radiology and Radiotherapy of Fujian Province (Digestive, Hematological and Breast Malignancies), Fuzhou, 350001 China; 5 Department of Gastroenterology, Fujian Medical University Union Hospital, Xinquan Road 29, Fuzhou, 350001 China; 6 Fujian Medical University Cancer Center, Fujian Medical University, Fuzhou, 350001 China; 7 Department of Gastrointestinal Surgery, Fujian Medical University Union Hospital, Fuzhou, 350001 China; 8 Department of Radiation Oncology, The Second Hospital of Zhangzhou, Zhangzhou, 363100 China; 9 Department of Medical Imagine Technology, College of Medical Technology and Engineering, Fujian Medical University, Fuzhou, 350001 China

**Keywords:** Rectal cancer, Oxaliplatin, Capecitabine, Chemoradiotherapy

## Abstract

**Background:**

Distant metastasis has been the main failure pattern for locoregionally advanced rectal cancer (LARC) patients, and intensified neoadjuvant chemotherapy has become a popular research topic. The present study aimed to compare the survival outcomes, acute toxicities and surgical complications in LARC patients who received preoperative chemoradiotherapy with triweekly oxaliplatin and capecitabine (triweekly XELOX) or capecitabine.

Methods: Between 2007 and 2017, patients with clinically staged II-III rectal cancer who were treated with preoperative chemoradiotherapy using either triweekly XELOX (oxaliplatin 130 mg/m^2^ plus capecitabine 825 mg/m^2^) or capecitabine were included. Variables potentially influencing chemotherapy treatment selection were used to generate propensity scores (PS). The association between chemotherapy regimens and survival endpoints, including distant metastasis-free survival (DMFS), overall survival (OS) and disease-free survival (DFS), were evaluated and adjusted with PS. The acute toxicities and surgical complications were also compared.

**Results:**

A total of 810 patients were included in the analysis; 277 (34.2%) patients received triweekly XELOX, and 533 (65.8%) received capecitabine. The pathological complete response (pCR) rates were 20.2 and 19.9% (*P* = 0.912) for the groups treated with triweekly XELOX and capecitabine, respectively. The 5-year DMFS, OS and DFS with triweekly XELOX versus capecitabine were 75.6% vs. 77.6% (*P* = 0.555), 79.2% vs. 83.3% (*P* = 0.101), and 69.9% vs. 73.7% (*P* = 0.283), respectively. Triweekly XELOX was not associated with an increased risk of severe toxicity during chemoradiotherapy, but it increased the risk of postoperative complications compared to capecitabine. After PS adjustment, the differences between the two groups remained insignificant in pCR rate, survival outcomes, and acute toxicities, and the difference in surgical complications disappeared.

**Conclusions:**

Triweekly XELOX or capecitabine concurrent with neoadjuvant radiotherapy leads to similar long-term survival outcomes, acute toxicities and surgical complications in LARC patients.

**Supplementary Information:**

The online version contains supplementary material available at 10.1186/s12885-022-09855-z.

## Background

Fluorouracil-based preoperative chemoradiotherapy (CRT) followed by total mesorectal excision (TME) is the standard option for the initial treatment of locally advanced rectal cancer (LARC) in terms of downstaging to pathological complete response, increasing rates of sphincter-saving surgery and decreasing local recurrence [1–3]. However, this improvement in local control has not been paralleled by an increase in long-term overall survival (OS), largely owing to the persistently high rate of distant metastasis (29–39%) [2, 4]. Thus, it makes sense to achieve better control of systemic disease for longer survival.

Borrowing from the survival benefits of adding oxaliplatin to fluorouracil-based adjuvant chemotherapy in colon cancer [5–7], several large randomized trials have tested the combination in LARC, concomitantly with neoadjuvant radiotherapy. Apart from Jiao’s study [8] and the German CAO/ARO/AIO-04 trial [9], which reported improvements in distant metastasis-free survival (DMFS) and disease-free survival (DFS), respectively, most studies have failed to show survival benefits with the addition of oxaliplatin [10–13]. The unaltered survival implies that low-dose weekly oxaliplatin was unable to further eliminate tumour micrometastasis, providing a rationale for intensified systemic treatment. Our previous study and Gao et al. reported that oxaliplatin given triweekly at a dose of 130 mg/m^2^ added to capecitabine (triweekly XELOX) concomitant with preoperative radiotherapy was tolerable and associated with excellent compliance, as well as promising long-term outcomes [14, 15]. However, the reports were both based on small sample sizes, and no clear evidence exists for the superiority of triweekly XELOX compared to capecitabine alone with preoperative chemoradiotherapy in patients with LARC.

Therefore, we focused on the comparison of long-term survival outcomes and the adverse effects of capecitabine plus neoadjuvant radiation with or without triweekly oxaliplatin in LARC patients. Our findings could help to guide clinical decision-making regarding neoadjuvant chemoradiotherapy treatment strategies in LARC.

## Methods

### Patient selection

In this unicentral, retrospective, noninterventional study, we included patients with histologically diagnosed rectal cancer of clinical stages II-III treated at our hospital between September 2007 and October 2017. All patients were treated with concurrent chemoradiotherapy followed by definitive surgery. Patients with metastatic or recurrent rectal cancer or patients who underwent endoscopic mucosal resection or transanal local excision were excluded. Patients with nonmetastatic disease who were found to have metastasis in the reassessment before surgery or during surgery were also excluded to avoid misdiagnosis in the first assessment.

The retrospective study was approved by the Clinical Research Ethics Committee of the Fujian Medical University Union Hospital, and due to the retrospective nature of the study, the requirement for informed consent was waived. The study was performed in accordance with the Declaration of Helsinki.

### Staging

Before treatment, patients underwent a routine staging procedure consisting of physical examination, digital rectal examination, colonoscopy, chest computed tomography, abdominal and pelvic magnetic resonance imaging or positron emission tomography-computed tomography scan. Routine laboratory tests consisted of complete blood count and chemistry, including renal function. Clinical stage was assessed according to the 7th edition of the International Union against Cancer/American Joint Committee on Cancer (UICC/AJCC) staging system.

### Chemotherapy

The concurrent chemotherapy consisted of capecitabine alone or double chemotherapy with oxaliplatin and capecitabine. In the capecitabine alone group, patients received oral capecitabine 825 mg/m^2^ twice daily during radiotherapy. In the double chemotherapy group (triweekly XELOX), patients were administered 130 mg/m^2^ intravenous oxaliplatin on Day 1 plus 825 mg/m^2^ capecitabine twice daily from Days 1–14 every 21 days. The selection of the concurrent chemotherapy regimen mainly depended on the experiences of the surgeon and oncologist and patient preference, with some consideration of the economic situation of the patients.

Postoperative adjuvant chemotherapy was determined by the treating surgeon and the patient. Most adjuvant chemotherapy was an oxaliplatin-based regimen, and only two patients received capecitabine alone.

### Radiotherapy

All patients received preoperative radiotherapy with either 3-dimensional conformal radiotherapy (3D-CRT) or intensity-modulated radiotherapy (IMRT). Radiation treatment planning was designed in accordance with previous studies conducted at the Fujian Medical University Union Hospital [14, 16]. Briefly, radiotherapy was delivered at 1.8 to 2.0 Gy (Gy) daily Monday through Friday for a total of 25 to 28 fractions over 5 to 6 weeks and a total dose of 45 Gy to 50.4 Gy. Radiation was delivered with a minimum energy of 6-MV photons through a three-field or five-field technique to the primary tumour and to mesorectal, presacral, and internal iliac lymph node drainage regions.

### Surgery

Radical surgery according to the TME principle was performed for all patients. Pathological stage was determined based on the surgical specimen. Primary tumour downstaging was determined by comparing the pathologic T stage with the baseline clinical T stage, and a pathologic T stage lower than the baseline clinical T stage was considered tumour downstaging. A pathological complete response (pCR, ypT0N0) was defined as the complete absence of tumour cells at the primary site and without lymph node involvement. The rectal cancer regression grade (RCRG) after preoperative treatment was evaluated according to Wheelers’ classification [17]. The RCRG classification groups were as follows: RCRG1, sterilization or only microscopic foci of adenocarcinoma remaining, with marked fibrosis; RCRG2, marked fibrosis but macroscopic disease present; RCRG3, little or no fibrosis, with abundant macroscopic disease.

### Statistical analysis

OS was calculated as the time from the first day of treatment to death from any cause or the last follow-up. DFS was defined as the time from the date of commencement of treatment to the first occurrence of any of the following events: local and/or regional recurrence, distant metastasis, or death from any cause. DMFS was defined as the time from the commencement of treatment to the date of metastasis. During therapy, adverse events were evaluated according to National Cancer Institute Common Terminology Criteria, version 4.0.

Baseline and clinical characteristics were used to examine balance between groups using absolute standardized mean differences (SMD), which describe between-group differences in units of SMD and are not substantially influenced by sample size in large cohorts [18]. Differences greater than 0.1 were considered clinically meaningful [18]. Survival was assessed using the Kaplan-Meier method, and categorical variables were compared using the log-rank test. The independent prognostic factors for rectal cancer were identified by the Cox proportional hazard regression model. To control for the misdistribution of the treatment period, we calculated the survival outcomes by comparing the triweekly XELOX cohort to the capecitabine cohort treated during 2012–2017. To focus on patients (clinical non lymph node metastasis (cN-) and pathological ypStage I-II) who likely benefited from the addition of oxaliplatin reported in the CAO/ARO/AIO-04 trial [9], we performed sensitivity analyses in the subgroups mentioned above.

An unadjusted comparison between patients receiving capecitabine and triweekly XELOX would be prone to bias. To enable a comparison in equivalent groups, a 1:2 propensity score (PS) analysis was performed between the two patient cohorts [18–21]. The propensity score was calculated for each patient using logistic regression with the variable that had a potential confounding effect, such as age at diagnosis, sex, interval from end of radiotherapy (RT) to surgery, clinical T stage, clinical N stage, RT method, and adjuvant chemotherapy. A PS-matched cohort was then created, yielding a cohort of 510 patients. PS-adjusted Cox regression models were used to evaluate the associations of concurrent chemotherapy regimens with treatment outcomes.

Statistical analyses were performed using SPSS software, version 23.0 (2015 IBM Corporation, Armonk, NY, USA), and the *R* programming language (*R* version 4.1.0). All tests used to explore statistical significance were 2 sided, and *P* < 0.05 was considered statistically significant.

## Results

### Patient characteristics

**Fig. 1:**
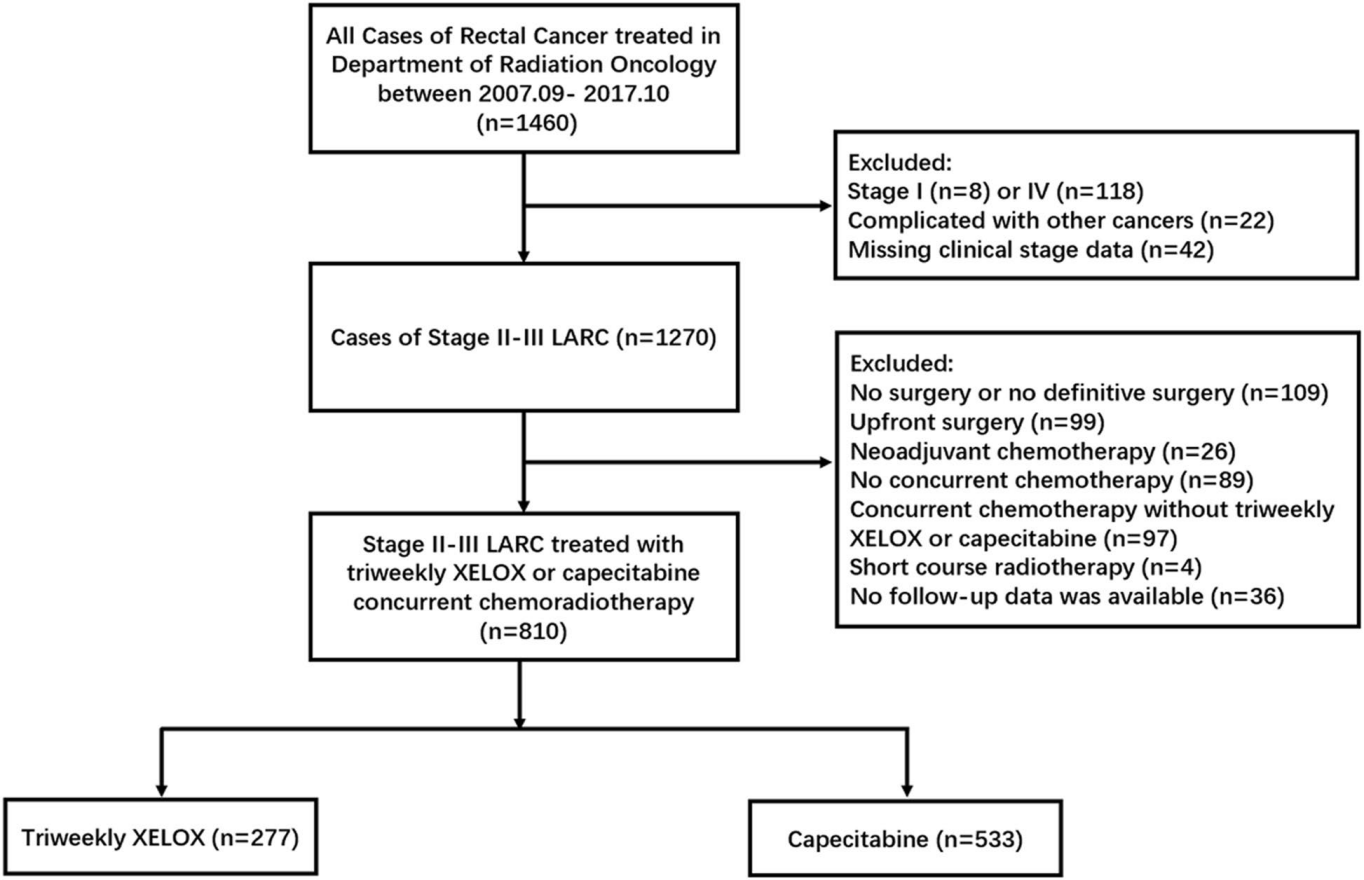
Included and excluded patients with rectal cancer

See Fig. 1 for a CONSORT diagram illustrating how we arrived at the analytic sample of 810 patients. From September 2007 to October 2017, 1477 patients were screened, and 810 patients met the eligibility criteria, of whom 277 (34.2%) were treated with triweekly XELOX and 533 (65.8%) with capecitabine. The distribution of baseline characteristics is reported in Table 1. In the triweekly XELOX group, 108 (39.0%) patients were treated in the early years (2007–2011), while only 2 patients (0.4%) in the capecitabine group were treated during the period (SMD = − 0.792). Regarding radiotherapy, 254 (91.7%) patients in the triweekly XELOX group and 525 (98.5%) patients in the capecitabine group received a dose of 50 or 50.4 Gy. The use of IMRT increased over time, so more patients in the capecitabine group were treated with IMRT (SMD = − 0.638). Patients receiving triweekly XELOX were younger (SMD = − 0.259) and tended to have a lower clinical T stage (SMD = − 0.145). After 1:2 matching based on PS, a total of 510 patients were included for subsequent analyses. Most variables were similarly distributed between the two groups after PS matching, except for the treatment period and the radiotherapy dose (Table 1).

**Table 1:** Baseline characteristics of the rectal cancer patients before and after propensity score matching

	Baseline			After propensity score matching		
	Triweekly XELOX ***n*** = 277	Capecitabine ***n*** = 533	SMD	TriweeklyXELOX ***n*** = 191	Capecitabine ***n*** = 319	SMD
**Age (years) median (range)**	55 (24–79)	58 (24–84)	− 0.259	56 (24–79)	56 (24–84)	0.006
**Gender**						
**Male**	184 (66.4%)	346 (64.9%)		124 (64.9%)	201 (63.0%)	
**Female**	93 (33.6%)	187 (35.1%)	0.032	67 (35.1%)	118 (37.0%)	0.040
**Treatment period**						
**2007–2011**	108 (39.0%)	2 (0.4%)		52 (27.2%)	2 (0.6%)	
**2012–2017**	169 (61.0%)	531 (99.6%)	−0.792	139 (72.8%)	317 (99.4%)	−0.598
**Clinical stage**						
**II**	29 (10.5%)	34 (6.4%)		15 (7.9%)	26 (8.2%)	
**III**	248 (89.5%)	499 (93.6%)	−0.133	176 (92.1%)	293 (91.8%)	0.011
**cT stage**						
**cT2**	25 (9.0%)	10 (1.9%)		8 (4.2%)	6 (1.9%)	
**cT3**	102 (36.8%)	222 (41.7%)		73 (38.2%)	136 (42.6%)	
**cT4**	150 (54.2%)	301 (56.5%)	−0.145	110 (57.6%)	177 (55.5%)	−0.004
**cN stage**						
**cN-**	29 (10.5%)	34 (6.4%)		15 (7.9%)	26 (8.2%)	
**cN+**	248 (89.5%)	499 (93.6%)	−0.134	176 (92.1%)	293 (91.8%)	0.011
**Type of Radiation**						
**3D-CRT**	134 (48.4%)	88 (16.5%)		50 (26.2%)	77 (24.1%)	
**IMRT**	143 (51.6%)	445 (83.5%)	−0.638	141 (73.8%)	242 (75.9%)	−0.046
**RT dose**						
**< 45 Gy**	1 (0.4%)	0 (0.0%)		1 (0.5%)	0 (0.0%)	
**45Gy–50Gy**	22 (7.9%)	8 (1.5%)		10 (5.2%)	4 (1.3%)	
**50Gy/50.4 Gy**	254 (91.7%)	525 (98.5%)	−0.370	180 (94.2%)	315 (98.7%)	−0.305
**Interval from RT to surgery (weeks), median (range)**	8 (4–20)	9 (4.3–19.9)	−0.543	8.6 (3.9–20)	9 (4.3–19.9)	−0.107
**Type of Surgery**						
**Abdominoperineal resection**	29 (10.5%)	58 (10.9%)		21 (11.0%)	35 (11.0%)	
**Anterior resection**	240 (86.6%)	468 (87.8%)		165 (86.4%)	280 (87.8%)	
**Other**	8 (2.9%)	7 (1.3%)	0.056	5 (2.6%)	4 (1.3%)	0.037
**Adjuvant chemotherapy**						
**Yes**	226 (81.6%)	404 (75.8%)		149 (78.0%)	243 (76.2%)	
**No**	51 (18.4%)	129 (24.2%)	0.149	42 (22.0%)	76 (23.8%)	0.044

*SMD* Standardized mean difference, *RT* Radiotherapy, *3D-CRT* 3-dimensional conformal radiotherapy, *IMRT* Intensity modulated radiotherapy

### Surgery and pathological findings

In the unadjusted cohort, the median time between surgery and chemoradiotherapy was 8 weeks (range, 4–20 weeks) for the triweekly XELOX group and 9 weeks (range, 4.3–19.9 weeks) for the capecitabine group (SMD = − 0.543) (Table 1). Anterior resection was the most common surgery type. The postoperative pathological findings are presented in Table 2. The pCR rate did not differ between the two groups (20.2% vs. 19.9% in the triweekly XELOX group and capecitabine group, respectively, SMD = 0.008). Regarding concerning primary tumour downstaging, it occurred less often in the triweekly XELOX group than in the capecitabine group (69.7% vs. 77.7%, SMD = 0.174). After PS adjustment, the difference in primary downstaging between the triweekly XELOX group and the capecitabine group disappeared (71.7% vs. 78.1%, SMD = − 0.101, Table 2). There was also no significant difference in the pCR rate (SMD = 0.037), ypT stage (SMD = 0.069), ypN stage (SMD = 0.073) or RCRG grade (SMD = − 0.096) in the PS-matched cohort. There were more lymph nodes retrieved in the capecitabine group than in the triweekly XELOX group (11 vs. 13, SMD = − 0.291). Survival analyses showed that dissection of ≥12 lymph nodes was not significantly associated with DMFS, DFS or OS in the unadjusted cohort (Additional Figure 1) or in the PS-matched cohort (Additional Figure 2).

**Table 2:** Pathologic characteristics of the rectal cancer patients

	Baseline			After propensity score matching		
	Triweekly XELOX ***n*** = 277	Capecitabine ***n*** = 533	SMD	Triweekly XELOX ***n*** = 191	Capecitabine ***n*** = 319	SMD
**ypT stage**						
**ypT0**	60 (21.7%)	111 (20.8%)		43 (22.5%)	66 (20.7%)	
**ypT1–2**	73 (26.4%)	179 (33.6%)		50 (26.2%)	113 (35.4%)	
**ypT3–4**	144 (52.0%)	243 (45.6%)	0.069	98 (51.3%)	140 (43.9%)	0.069
**ypN stage**						
**ypN0**	186 (67.1%)	395 (74.1%)		133 (69.6%)	231 (72.4%)	
**ypN1**	70 (25.3%)	113 (21.2%)		46 (24.1%)	73 (22.9%)	
**ypN2**	21 (7.6%)	25 (4.7%)	0.157	12 (6.3%)	15 (4.7%)	0.073
**No. of sampled lymph nodes**						
**Median (range)**	11 (1–88)	13 (0–73)	−0.094	11 (1–34)	13 (0–73)	− 0.291
**ypStage**						
**0**	57 (20.6%)	106 (19.9%)		41 (21.5%)	62 (19.4%)	
**I**	57 (20.6%)	145 (27.2%)		41 (21.5%)	91 (28.5%)	
**II**	70 (25.3%)	142 (26.6%)		49 (25.7%)	78 (24.5%)	
**III**	89 (32.1%)	131 (24.6%)		57 (29.8%)	83 (26.0%)	
**IV**	4 (1.4%)	9 (1.7%)	0.106	3 (1.6%)	5 (1.6%)	0.059
**Primary tumor downstaging**						
**Yes**	193 (69.7%)	414 (77.7%)		137 (71.7%)	249 (78.1%)	
**No**	84 (30.3%)	119 (22.3%)	0.174	54 (28.3%)	70 (21.9%)	−0.101
**pCR**						
**Yes**	56 (20.2%)	106 (19.9%)		40 (20.9%)	62 (19.4%)	
**No**	221 (79.8%)	427 (80.1%)	0.008	151 (79.1%)	257 (80.6%)	0.037
**RCRG**						
**1**	139 (50.2%)	283 (53.1%)		95 (49.7%)	167 (52.4%)	
**2**	82 (29.6%)	210 (39.4%)		68 (35.6%)	128 (40.1%)	
**3**	11 (4.0%)	35 (6.6%)	0.140	7 (3.7%)	21 (6.6%)	−0.096
**Undermined**	45 (16.2%)	5 (0.9%)		21 (11.0%)	3 (0.9%)	

*SMD* Standardized mean difference, *No*. Number, *pCR* Pathological complete response, *RCRG* Rectal cancer regression grade

### Survival outcomes and prognostic factors

**Fig. 2:**
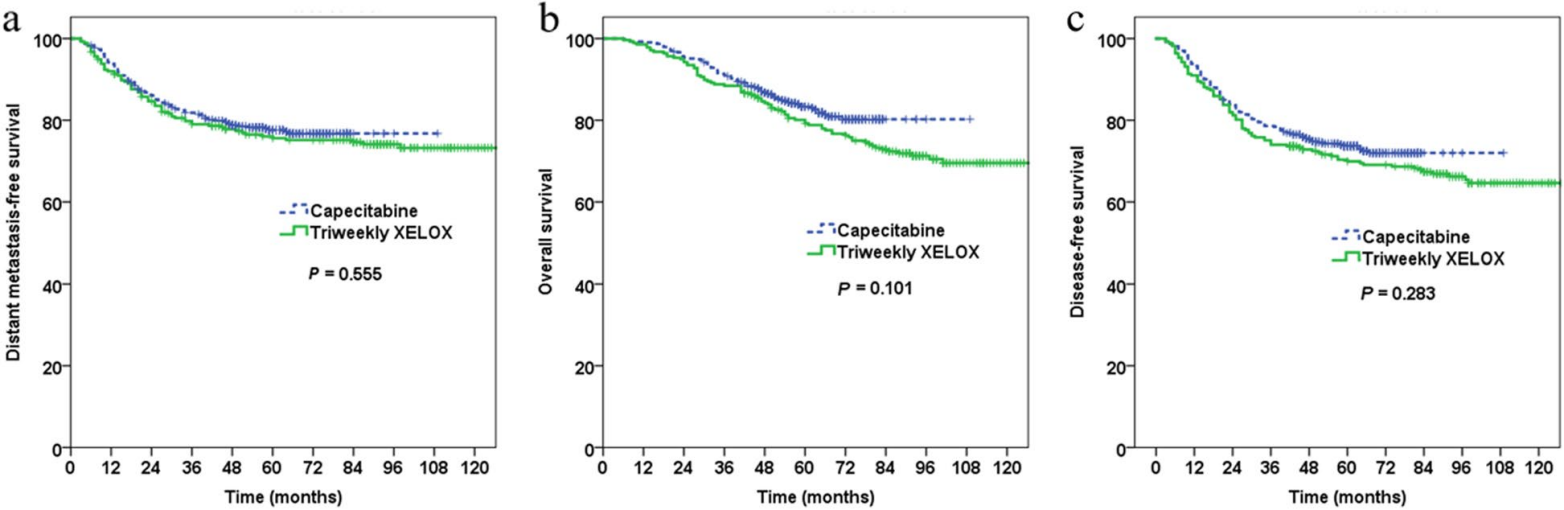
Unadjusted survival curves of all patients by treatment with triweekly XELOX vs. capecitabine. **a** Unadjusted analysis of distant metastasis-free survival; **b** Unadjusted analysis of overall survival; **c** Unadjusted analysis of disease-free survival

**Fig. 3:**
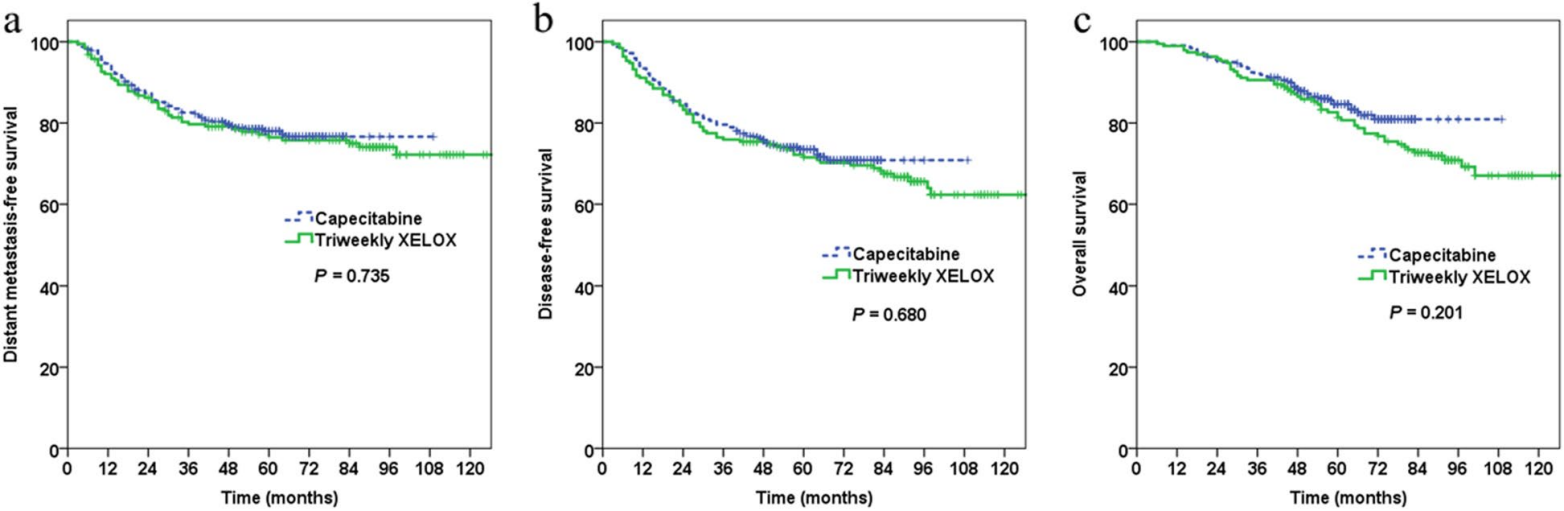
Propensity score (PS)-matched survival curves of patients by treatment with triweekly XELOX vs. capecitabine. **a** PS-matched analysis of distant metastasis-free survival; **b** PS-matched analysis of overall survival; **c** PS-matched analysis of disease-free survival

The median follow-up was 63 months (range, 6–152) for all patients and 89 months (range, 6–152) and 59 months (range, 6–109) for the triweekly XELOX group and capecitabine group, respectively. The 5-year DMFS, OS and DFS were 75.6% vs. 77.6% (*P* = 0.555), 79.2% vs. 83.3% (*P* = 0.101), and 69.9% vs. 73.7% (*P* = 0.283) for the patients treated with triweekly XELOX and capecitabine, respectively (Fig. 2). After PS adjustment, there remained no survival benefit to using triweekly XELOX in the PS-matched cohort (Fig. 3).

**Fig. 4:**
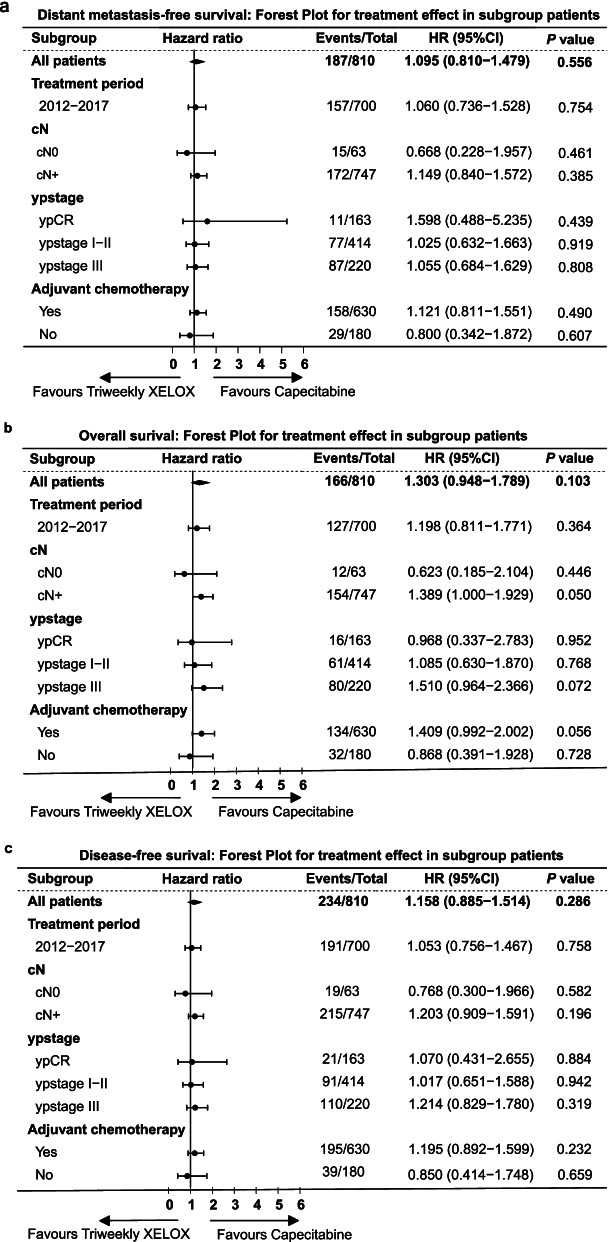
Forest plot for the effect of oxaliplatin on distant metastasis-free survival (**a**), overall survival (**b**) and disease-free survival (**c**) in subgroups patients

Considering that most patients in the capecitabine group were treated during 2012–2017, while the patients in the triweekly XELOX group were not, we repeated our survival analysis excluding patients treated during 2007–2011. We found similar survival outcomes in this analysis, including DMFS (*P* = 0.754), OS (*P* = 0.364) and DFS (*P* = 0.758) (Fig. 4). Triweekly XELOX was also not associated with improved DMFS, OS or DFS in the sensitivity analysis considering patients with different clinical cN stages and pathological ypStages or whether they received adjuvant chemotherapy (Fig. 4).

The results of multivariate analysis of the prognostic factors are shown in Table 3. In the whole cohort of patients, multivariate analysis showed that the regimen of concurrent chemotherapy had no effect on DMFS (*P* = 0.871), OS (*P* = 0.682) or DFS (0.820), while ypT stage and ypN stage were independent prognostic factors for DFS, OS and DFS (all *P* values < 0.001). In the PS-adjusted cohort, the regimen of concurrent chemotherapy was still not an independent prognostic factor for DMFS, OS, or DFS.

**Table 3:** Multivariable Cox regression analyses of variables correlated with clinical outcomes on the patients with locally advanced rectal cancer

	Distant metastasis-free survival		Overall survival		Disease-free survival	
	***P***	HR (95%CI)	***P***	HR (95%CI)	***P***	HR (95%CI)
**Unadjusted cohort**						
**Gender**	0.035*	0.729 (0.543–0.978)	0.348	0.859 (0.626–1.180)	0.144	0.820 (0.628–1.070)
**Age**	0.674	0.937 (0.693–1.267)	0.165	1.247 (0.913–1.702)	0.797	1.036 (0.792–1.355)
**cT stage**	0.500	0.918 (0.715–1.178)	0.757	0.959 (0.733–1.254)	0.929	1.010 (0.806–1.266)
**cN stage**	0.309	0.758 (0.445–1.292)	0.821	1.074 (0.580–1.986)	0.286	0.772 (0.480–1.242)
**Pretreatment CEA**	0.072	1.265 (0.979–1.634)	0.076	1.284 (0.975–1.691)	0.110	1.208 (0.958–1.523)
**Type of radiation**	0.673	1.073 (0.772–1.492)	0.836	0.962 (0.668–1.386)	0.849	0.970 (0.707–1.330)
**Concurrent chemotherapy**	0.871	0.973 (0.695–1.361)	0.682	1.073 (0.765–1.506)	0.820	0.967 (0.724–1.291)
**Adjuvant chemotherapy**	0.112	1.379 (0.927–2.052)	0.814	0.954 (0.641–1.418)	0.182	1.265 (0.896–1.786)
**pCR**	0.796	1.119 (0.477–2.626)	0.003*	3.634 (1.532–8.623)	0.160	1.648 (0.820–3.312)
**ypT stage**	< 0.001*	2.066 (1.598–2.670)	< 0.001*	1.902 (1.497–2.417)	< 0.001*	1.770 (1.431–2.188)
**ypN stage**	< 0.001*	1.768 (1.439–2.172)	< 0.001*	2.048 (1.639–2.558)	< 0.001*	1.941 (1.615–2.331)
**RCRG**	0.817	0.992 (0.923–1.066)	0.133	1.050 (0.985–1.120)	0.287	1.031 (0.975–1.091)
**PS-adjusted cohort**						
**Gender**	0.414	0.854 (0.584–1.248)	0.923	1.021 (0.666–1.565)	0.510	0.893 (0.636–1.252)
**Age**	0.856	1.036 (0.707–1.519)	0.029*	1.562 (1.047–2.330)	0.291	1.196 (0.858–1.668)
**cT stage**	0.629	0.921 (0.660–1.285)	0.492	1.138 (0.787–1.643)	0.591	1.086 (0.804–1.466)
**cN stage**	0.469	0.773 (0.385–1.551)	0.768	1.127 (0.508–2.500)	0.828	0.932 (0.494–1.758)
**Pretreatment CEA**	0.011*	1.526 (1.101–2.116)	0.013*	1.578 (1.100–2.263)	0.013*	1.447 (1.081–1.937)
**Type of radiation**	0.438	1.182 (0.775–1.802)	0.492	1.169 (0.749–1.825)	0.712	1.072 (0.743–1.546)
**Concurrent chemotherapy**	0.855	0.964–0.651-1.429)	0.482	1.163 (0.764–1.769)	0.775	0.950 (0.670–1.348)
**Adjuvant chemotherapy**	0.040*	1.771 (1.027–3.053)	0.919	1.027 (0.612–1.724)	0.194	1.338 (0.862–2.077)
**pCR**	0.383	0.623 (0.215–1.804)	0.256	1.864 (0.636–5.466)	0.960	1.022 (0.443–2.359)
**ypT stage**	0.001*	1.701 (1.240–2.334)	0.037*	1.430 (1.023–2.000)	0.005*	1.459 (1.121–1.899)
**ypN stage**	< 0.001*	1.903 (1.457–2.487)	< 0.001*	2.253 (1.680–3.022)	< 0.001*	2.023 (1.594–2.566)
**RCRG**	0.577	1.027 (0.935–1.129)	0.243	1.056 (0.964–1.157)	0.365	1.037 (0.959–1.122)

*HR* Hazard ratio, *CI* Confidence interval, *pCR* Pathological complete response, *RCRG* Rectal cancer regression grade, *PS* Propensity score. * *P* < 0.05

### Compliance and acute toxicity

In an unadjusted analysis, 99.2% of patients in the triweekly XELOX group completed two cycles of chemotherapy, and 99.6% patients in the triweekly XELOX group and 100% patients in the capecitabine group received at least 45Gy; in the former group, only 1 patient received a dose of 41.4 Gy/23F due to poor health and treatment toxicity, while no patients in the latter group did.

There was no incidence of treatment-related mortality. Grade 3–4 toxicities of any type during CRT were observed in 34 patients (12.3%) in the triweekly XELOX group and in 47 patients (8.8%) in the capecitabine group (*P* = 0.077). The most frequent toxicities were leukopenia, neutropenia and diarrhoea. In both the unadjusted analysis and PS-adjusted analysis, compared to capecitabine, triweekly XELOX did not increase the risk of severe acute toxicities during CRT (hazard ratio (HR) =1.447, 95% confidence interval (CI): 0.907–2.309, *P* = 0.121, and HR = 1.392, 95% CI: 0.760–2.550, *P* = 0.284, respectively) (Table 4).

**Table 4:** Hazard ratio of adverse events among patients treated with triweekly XELOX versus capecitabine

Toxicity	Unadjusted HR for toxicity with triweekly XELOX (95%CI)	***P*** value	PS-adjusted HR for toxicity with triweekly XELOX (95%CI)	***P*** value
**Grade 3–4 toxicities during CRT**				
**Any toxicity**	1.447 (0.907–2.309)	0.121	1.392 (0.760–2.550)	0.284
**Leukopenia**	1.880 (0.970–3.644)	0.061	1.857 (0.876–3.937)	0.107
**Neutropenia**	0.961 (0.383–2.410)	0.933	1.117 (0.391–3.189)	0.836
**Anemia**	1.928 (0.120–30.934)	0.643	1.121 (0.257–20.132)	0.996
**Diarrhea**	0.733 (0.370–1.451)	0.733	0.633 (0.222–1.803)	0.392
**Postoperative complications**				
**Any complication**	1.574 (1.034–2.316)	0.034*	1.477 (0.882–2.472)	0.138
**Anastomotic leakage**	1.072 (0.488–2.354)	0.863	0.830 (0.306–2.248)	0.714
**Incision infection**	1.533 (0.686–3.423)	0.297	2.745 (0.885–8.517)	0.080
**Urinary tract infection**	1.101 (0.320–3.794)	0.879	0.832 (2.06–3.368)	0.797
**Pulmonary infection**	1.850 (0.901–3.800)	0.094	1.563 (0.676–3.616)	0.296
**Bowel obstruction**	3.403 (1.324–8.744)	0.011*	2.554 (0.711–9.169)	0.150
**Abdominal or pelvic infection**	0.725 (0.317–1.659%)	0.446	0.661 (0.204–2.137)	0.489

*HR* Hazard ratio, *CI* Confidence interval, *PS* Propensity score, *CRT* Chemoradiotherapy. * *P* < 0.05

The total rate of postoperative complications was 17.7% (49/277) with triweekly XELOX versus 12.2% (65/533) with capecitabine (*P* = 0.033). Pulmonary infection (5.4%) and bowel obstruction (4.3%) were more common with the triweekly XELOX regimen, whereas abdominal or pelvic infection (3.9%) was more frequent with the capecitabine regimen. Triweekly XELOX increased the risk of postoperative complications (HR = 1.574, 95% CI: 1.034–2.316, *P* = 0.034) in the unadjusted cohort (Table 4). After PS adjustment, the difference disappeared.

## Discussion

Oxaliplatin is a common regimen added to capecitabine in the preoperative treatment of rectal cancer; however, the clinical value of oxaliplatin remains controversial. Several randomized clinical trials have attempted to compare capecitabine (fluorouracil) with or without oxaliplatin for preoperative chemoradiotherapy of LARC, and they have shown that double regimens did not improve tumour response or patient survival [10, 11, 13, 22]. A meta-analysis by Hoerndervangers reported that the addition of oxaliplatin might result in more pCR, but this benefit does not translate into less local recurrence or improved survival [23]. Notably, the oxaliplatin mentioned above was administered weekly at a low dose of 50 mg/m^2^, which was thought to act as a radiation sensitizer. Fluorouracil-based chemoradiotherapy and optimal TME surgery have already maximized local tumour control to more than 90% [2, 4, 9], and there is little or no room for further improvement with the incorporation of additional radiosensitizing agents. Distant metastasis becomes the main failure. CAO/ARO/AIO/04 showed that the gain of chemotherapy seen in the risk of distant metastasis occurred early in the course of treatment [9]. Hence, it could naturally be inferred that a full dose of oxaliplatin (130 mg/m^2^/triweekly), which has a different biologic mechanism compared to a low dose of oxaliplatin (50–60 mg/m^2^/weekly), combined with capecitabine during preoperative chemoradiotherapy could ameliorate long-term outcomes.

Our study was the first to compare the triweekly XELOX regimen with the capecitabine regimen during preoperative chemoradiotherapy for LARC. A previous study reported that an intensified full dose of XELOX concomitant with preoperative radiotherapy provided a promising long-term oncologic outcome for LARC patients, with 5-year DFS and OS of approximately 80% [14, 15]. In the present study, compared to the capecitabine regimen, intensified chemotherapy with triweekly XELOX offered no statistically significant survival advantage. The pathological responses to preoperative chemoradiotherapy were also equally distributed among patients treated with or without oxaliplatin, excluding even minor and/or qualitative effects. Sensitivity analysis confirmed the consistent results that no survival benefits were observed with the use of triweekly XELOX in patients treated with adjuvant chemotherapy or patients treated during 2012–2017. CAO/ARO/AIO/04 showed that the benefit of adding oxaliplatin was observed in patients with clinical cN- rather than cN1–2 and in patients with pathological stage I and II disease, rather than ypCR or stage III disease [9]. We performed sensitivity analysis in the subgroup patients mentioned above, and still not found survival improvement with the use of triweekly XELOX.

The results were unexpected. Theoretically, intensified treatment would further downstage the tumour and nodal disease prior to surgery and/or target potential micrometastatic disease [23, 24]. STAR 01 reported a lower frequency of extrapelvic metastases found at surgery in patients treated with oxaliplatin [10]. Jiao et al. reported that adding oxaliplatin to capecitabine-based preoperative chemoradiotherapy could significantly reduce metastasis by 11.66% (*P* = 0.045) [8]. Meta-analyses provided further evidence for the application of oxaliplatin [25, 26]. One possible reason for the negative results of the current study was that the number of retrieved lymph nodes in the triweekly XELOX group was significantly smaller than that in the capecitabine group (11 vs. 13, SMD = − 0.291). A greater number of nodes increases the likelihood of proper staging [27, 28]. Some patients who might benefit from adjuvant therapy are misclassified as node-negative due to incomplete sampling of lymph nodes [29]. Kenjiro Kotake et al. reported that, with one increase in the number of lymph nodes retrieved, the mortality risk decreased by 2.1% for Stage II and by 0.8% for Stage III [30]. Previous studies have also reported that the number of retrieved lymph nodes was an important prognostic factor [31, 32], although in the present study, examination of at least 12 lymph nodes did not significantly improve survival. The detection of lymph nodes was associated with the experience of the surgeon and the pathologist, as well as the preoperative treatment. Neoadjuvant therapy can lead to a significantly decreased number of lymph nodes detected due to radiation-induced fibrosis, lymphocyte depletion, tissue contraction, adipocyte replacement and interstitial atrophy, making it more difficult to detect lymph nodes during surgery or pathological examination [33, 34]. Previous criteria for dissecting at least 12 lymph nodes might not be suitable for patients who receive neoadjuvant CRT. The minimal number of lymph nodes for patients who receive neoadjuvant CRT has still acquired a consensus. Further large trials are needed to validate the effects of retrieved lymph nodes. Another possible reason was that the treatment era was different between the two groups. A total of 27.2% of patients in the triweekly XELOX group received treatment in the earlier years (2007–2011), while only 0.6% of patients in the capecitabine group were treated at that time. This difference might somewhat offset the benefits of triweekly XELOX since the technique level of radiotherapy and surgery evolved over time. The FOWARC trial conducted in China investigated the addition of relatively high-dose oxaliplatin (85 mg/m^2^) to neoadjuvant fluorouracil-based chemoradiotherapy and reported similar results; no improvement in DFS or OS was shown among patients with stage II to III rectal cancer despite a higher pCR rate [35].

The cumulative dose of oxaliplatin has been reported to affect patient survival. Chang et al. reported that patients with a cumulative dose of oxaliplatin less than 460 mg/m^2^ had poorer OS, DMFS and DFS [36]. The CAO/ARO/AIO-04 trial added oxaliplatin both to preoperative chemoradiotherapy and adjuvant chemotherapy, had a high cumulative dose of oxaliplatin of 1000 mg/m^2^, and significantly increased both the pCR rate and 3-year DFS by a rate of 4% [9]. Similarly, a trial by Jiao et al. and the ADORE trial administered cumulative doses of oxaliplatin with 680 mg/m^2^ and 750–920 mg/m^2^, respectively, showing superiority of adding the oxaliplatin regimen in reducing distant metastasis and ameliorating DFS at 3 years [8, 37]. The trials that reported negative results had a relatively low cumulative dose of oxaliplatin (STAR-01360 mg/m^2^, ACCORD12 250 mg/m^2^ and NSABP R-04250 mg/m^2^). In our study, the cumulative dose of oxaliplatin in the triweekly XELOX group was similar to that in the ACCORD12 and NASABP-04 l trials (260 mg/m^2^), inducing consistently negative results with these trials.

To achieve better tumour control, oncologists have been exploring new neoadjuvant treatment strategies. An alternative strategy known as total neoadjuvant therapy was deemed to be a promising strategy in LARC [38]. Two recently reported phase III, randomized trials, PRODIGE 23 [39] and RAPIDO [40], reported that adding neoadjuvant chemotherapy to either standard short-course radiation or standard long-course chemoradiation in LARC patients reduced the risk of metastasis and significantly delayed disease-related treatment failure and increased disease-free survival. In an attempt to intensify neoadjuvant treatment, additional agents, such as bevacizumab, cetuximab, and veliparib, were also tested but failed to achieve their primary endpoints or led to higher rates of postoperative complications [41].

In both the full cohort and the PS-adjusted cohort, the addition of oxaliplatin did not significantly increase the risks of severe acute toxicities during CRT or postoperative complications. In our study, the incidence of grade 3–4 toxicities was very low (2.3%) in the triweekly XELOX group. In contrast, previous studies have shown that the addition of oxaliplatin to preoperative chemoradiotherapy significantly worsened toxicity, with grade 3–4 toxicity rates of up to 21.4–49.1% [8–10, 23]. The difference between our study and previous studies was that the oxaliplatin used in the previous studies was a weekly regimen for 5–6 cycles. This regimen suggested that oxaliplatin administered triweekly for 2 cycles was less toxic and more tolerable than the weekly strategy. Triweekly regimen administration compared to weekly regimen administration has been reported to reduce toxicities for several chemotherapeutic drugs, such as triweekly cisplatin in head and neck cancer and triweekly paclitaxel in breast cancer [42, 43]. A possible explanation was that, compared to the weekly dose-dense administration, there was a longer time free from chemotherapy in the triweekly regimen treatment strategy. During this period, patients could recover from the side effects.

The major deficiency of this study is its retrospective nature. Considerable heterogeneity between patients undergoing triweekly XELOX or standard capecitabine therapy could have influenced both primary outcomes. To control for bias, we performed PS analysis, and comparison of baseline characteristics showed that the patients in the two groups were comparable. Another limitation was that the time of follow-up was not sufficient in the capecitabine group. Considering the favourable prognosis of rectal cancer, further follow-up is needed to fully evaluate the long-term survival and late toxicities.

## Conclusions

Although not conclusive, our study showed that neoadjuvant chemoradiotherapy with triweekly XELOX did not improve pathological response or long-term outcomes relative to capecitabine alone in locally advanced rectal cancer. Adding the full dose of oxaliplatin to capecitabine did not increase acute toxicities or postoperative complications. A large, randomized, phase III trial is warranted to confirm the safety and efficacy of the triweekly XELOX regimen.

## Supplementary Information

**Additional file 1: Figure 1.** Unadjusted survival curves of all patients when the cutoff value of the total number of lymph nodes retrieved was 12. a) Unadjusted analysis of distant metastasis-free survival; b) Unadjusted analysis of disease-free survival; c) Unadjusted analysis of overall survival. **Figure 2.** Propensity score (PS)-matched survival curves of patients when the cutoff value of the total number of lymph nodes retrieved was 12. a) Unadjusted analysis of distant metastasis-free survival; b) Unadjusted analysis of disease-free survival; c) Unadjusted analysis of overall survival.

**Additional file 2.** Original data.

## Data Availability

All data generated or analysed during this study are included in the supplementary information files.

## Abbreviations

LARC: Locoregionally advanced rectal cancer
XELOX: Oxaliplatin and capecitabine
PS: Propensity scores
DMFS: Distant metastasis-free survival
OS: Overall survival
DFS: Disease-free survival
pCR: Pathological complete response
CRT: Chemoradiotherapy
TME: Total mesorectal excision
UICC/AJCC: Union against Cancer/American Joint Committee on Cancer
3D-CRT: 3-dimensional conformal radiotherapy
IMRT: Intensity modulated radiotherapy
Gray: Gy
RCRG: Rectal cancer regression grade
RT: Radiotherapy
HR: Hazard ratio
CI: Confidence interval
SMD: Standardized mean difference
