# Supplementary material for: Preoperative chemoradiotherapy with capecitabine and triweekly oxaliplatin versus capecitabine monotherapy for locally advanced rectal cancer: a propensity-score matched study
Source: BMC Cancer. 2022 Jul 18;22:789. doi: 10.1186/s12885-022-09855-z (PMC9295262; doi:10.1186/s12885-022-09855-z)
Supplement: Supplementary file 1 — Additional file 1: Figure 1. Unadjusted survival curves of all patients when the cutoff value of the total number of lymph nodes retrieved was 12. a) Unadjusted analysis of distant metastasis-free survival; b) Unadjusted analysis of disease-free survival; c) Unadjusted analysis of overall survival. Figure 2. Propensity score (PS)-matched survival curves of patients when the cutoff value of the total number of lymph nodes retrieved was 12. a) Unadjusted analysis of distant metastasis-free survival; b) Unadjusted analysis of disease-free survival; c) Unadjusted analysis of overall survival. [file 12885_2022_9855_MOESM1_ESM.docx]

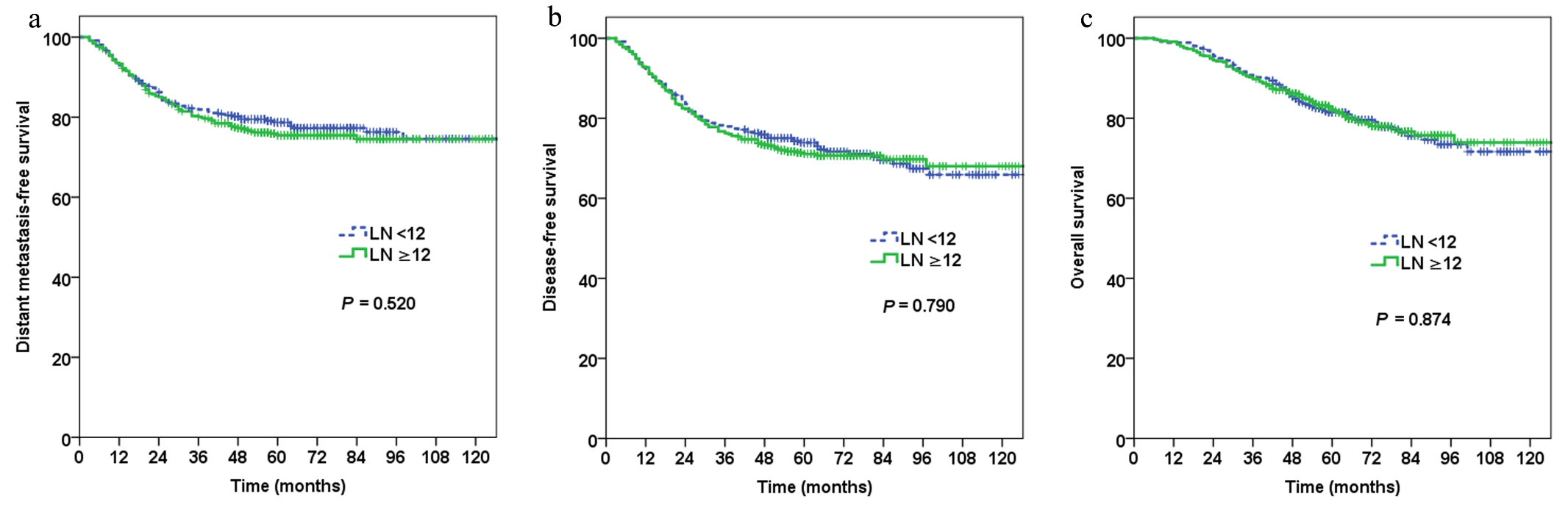


**Additional Figure 1.** Unadjusted survival curves of all patients when the cutoff value of the total number of lymph nodes retrieved was 12. a) Unadjusted analysis of distant metastasis-free survival; b) Unadjusted analysis of disease-free survival; c) Unadjusted analysis of overall survival.


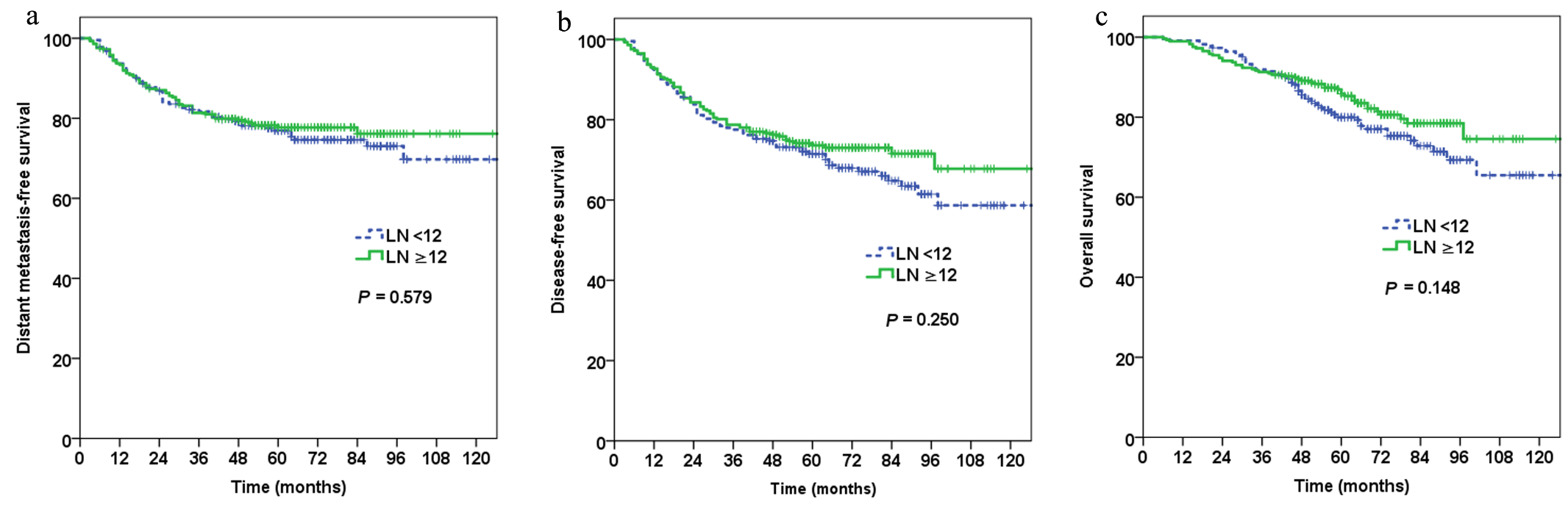


**Additional Figure 2.** Propensity score (PS)-matched survival curves of patients when the cutoff value of the total number of lymph nodes retrieved was 12. a) Unadjusted analysis of distant metastasis-free survival; b) Unadjusted analysis of disease-free survival; c) Unadjusted analysis of overall survival.
